# Needs of patients with dementia and their caregivers in primary care: lessons learned from the Alzheimer plan of Quebec

**DOI:** 10.1186/s12875-021-01528-3

**Published:** 2021-09-15

**Authors:** Vladimir Khanassov, Laura Rojas-Rozo, Rosa Sourial, Xin Qiang Yang, Isabelle Vedel

**Affiliations:** 1 Herzl Clinic, Jewish General Hospital and Department of Family Medicine, McGill University, Montreal, Canada; 2 Lady Davis Institute for Medical Research, Jewish General Hospital, Montreal, Canada; 3 Department of Family Medicine, McGill University, Montreal, Canada

**Keywords:** Dementia, primary healthcare, family medicine practice, needs, expectations, Quebec Alzheimer Plan

## Abstract

**Background:**

Persons living with dementia have various health and social care needs and expectations, some which are not fully met by health providers, including primary care clinicians. The Quebec Alzheimer plan, implemented in 2014, aimed to cover these needs, but there is no research on the effect this plan had on the needs and expectations of persons living with dementia. The objective of this study is to identify persons living with dementia and caregivers’ met and unmet needs and to describe their experience.

**Methods:**

This is a sequential mixed methods explanatory design: Phase 1: cross-sectional study to describe the met and unmet health and social care needs of community-dwelling persons living with dementia using *Camberwell Assessment of Need of the Elderly* and *Carers’ Assessment for Dementia* tools. Phase 2: qualitative descriptive study to explore and understand the experiences of persons living with dementia and caregivers with the use of social and healthcare services, using semi-structured interviews. Data from phase 1 was analyzed with descriptive statistics, and from phase 2, with inductive thematic analysis. Results from phases 1 and 2 were compared, contrasted and interpreted together.

**Results:**

The mean total number of needs reported by the patients was 5.03 (4.48 and 0.55 met and unmet needs, respectively). Caregivers had 0.52 met needs (3.16 unmet needs). The main needs for both were memory, physical health, eyesight/hearing/communication, medication, looking after home, money/budgeting. Three categories were mentioned by the participants: Persons living with dementia and caregiver’s attitude towards memory decline, their perception of community health services and of the family medicine practice.

**Conclusions:**

Our study confirms the findings of other studies on the most common unmet needs of the patients and caregivers that are met partially or not at all. In addition, the participants were satisfied with access to care, and medical services in primary practices, being confident in their family. Our results indicate persons living with dementia and their caregivers need a contact person, a clear explanation of their dementia diagnosis, a care plan, written information on available services, and support for the caregivers.

**Supplementary Information:**

The online version contains supplementary material available at 10.1186/s12875-021-01528-3.

## Background

Alzheimer’s disease and other related dementias has been recognized by the World Health Organization as the “global public health crisis of the 21st century” [1, 2]. Dementia is a neurodegenerative disorder characterized by decline in memory and cognitive functions that interfere with activities of daily living of individuals [3]. It is reported that every four seconds a new case of dementia is detected in the world (7.7 million new cases each year) [1].

### Needs of the patients living with dementia and their caregivers

Persons living with dementia (PWD) have various needs and expectations, some of them are not yet fully met (e.g., early diagnosis of dementia) [4]. The abbreviation of PWD is only used for the convenience of the article and to respect the word limits. Family caregivers are still the main source of help to this population and often report physical and psychological burden [5]. They are involved in the day-to-day care in addition to coordinating a variety of health services. One of the recent studies showed that more than 70% of caregivers had at least one unmet need that is underestimated by the healthcare system [6, 7].

### Role of primary care

PWD and their caregivers require a variety of interventions based on their health and social needs. However, a consistent and widespread criticism of the care received by PWD and their families in many countries is that it is fragmented and rarely person-centered [8]. There are different models of dementia patient-centered care but not routinely implemented in primary care (e.g., case management) [9–15].

Thus, it is imperative to restructure the healthcare system to deliver person-centered care able to address the multiple needs of this population.

A comprehensive systematic review has described the variety of the needs of PWD living in the community (e.g., assistance with daily activities, safety, access to FPs) and caregivers (emotional support, education on the diagnosis, advance care planning) as well as the impact of the intervention focused on the needs of these vulnerable populations – case management (e.g., better education on the disease, provision of sufficient information on dementia-specific community resources) [4].

Five Canadian consensus conferences on Alzheimer's disease have recommended that prevention-promotion, detection, diagnosis and treatment of patients should predominantly be under the responsibility of primary care [16, 17]. The best chance to address the health care demand driven by dementia is to get primary care involved early in the disease process [18]. However, the primary care system is not yet prepared to face the growing prevalence of this disease and to provide patient-centered care [19–22]. Thus, the majority of patients does not receive a diagnosis of dementia or the appropriate service in primary care [23]. In addition, the primary healthcare professionals tend to underestimate the impact of the disease on the caregivers [24]. In response, Canadian governments (e.g., Ontario [25], Quebec [26]) have developed their provincial programs.

The Quebec Ministry of Health implemented in 2014 the Quebec Alzheimer Plan [27] (hereafter, the Plan) in all Family Medicine Practices (FMP) [28]. According to the Plan, family physicians (FP) of FMP are responsible for the diagnosis of dementia, management of the memory decline and behavioral symptoms associated with dementia, treatment and support of caregivers [26]. FPs refer PWD to the specialized care services (e.g., memory clinic, geriatrics, neurology) when they need a co-management of complex cases or for an advice on other options of the treatment (e.g., poorly controlled behavioral symptoms, rapidly progressive memory decline, early onset of dementia). FPs work in close collaboration with a nurse (a case manager) who provides timely follow-ups, updates the treating physician on any change in the condition of PWD as well as connect the caregivers with the community support services (e.g., local Alzheimer society chapter). FPs and the nurses use a protocol on dementia investigation, pharmacologic and non-pharmacologic treatment and caregivers support [29, 30].

While there was no formal study focusing on the PWD and caregivers needs evaluation prior to the Plan implementation, a retrospective study on the impact on the Plan showed a significant improvement of the frequency and quality of follow-up of PWD after the Plan implementation [27].

Consequently, research is required to understand how the Plan has impacted the health and social response to the needs of PWD and their caregivers as well as their perceptions of the received services. Thus, the objective of this study is to identify PWDs’ and caregivers’ met and unmet needs and to describe their experience within the context of the Plan implementation.

## Methods

This is a sequential mixed methods explanatory design [31] composed of a cross-sectional quantitative study (Phase 1) to describe the met and unmet health and social care needs of community-dwelling PWD and their caregivers, followed by a qualitative descriptive study (Phase 2) to further explore and understand their experience. We integrated the quantitative and qualitative results to identify how the needs of PWD and their caregivers are met (or unmet).

### Participants and setting

This study was conducted in three FMPs in Montreal, Quebec. All three sites are the teaching facilities for the residents. FMPs provided a list of the patients with the diagnosis of dementia (code 08 as per provincial administrative data-base nomenclature). Based on the list, we identified persons with a diagnosis of dementia or mild cognitive impairment (Mini-Mental State Examination (MMSE) (score above 10) and the Montreal Cognitive Assessment (MoCA) (score above 3) as reported in the patient’s chart); able to speak French or English; community dwelling; who have an informal caregiver (caregiver hereafter); and who were able to consent, report feelings, and express concerns regarding the disease [32].. For the caregivers, we selected persons knowledgeable about the PWD, and who spent a minimum of 4 hours (unpaid) per day [33]; who were the primary contact in the patient’s medical record; and who routinely accompanies the PWD to the FP. All participants signed consent forms for each phase of the study. We recruited a total of 29 PWD and 25 caregivers for Phase 1, of which 7 dyads (14 participants) participated in Phase 2. The drop-off in recruitment was due to unforeseen circumstances (the COVID-19 pandemics). We recruited participants and collected data between November 2018 and January 2020.

### Recruitment

Our recruitment strategy was composed of several steps involving the FMPs and the research team (See online Additional file 1 for more information).

For Phase 1:

- At each FMP, a nurse was in charge of requesting the FPs a list of the potential participants who correspond to the eligibility criteria stated above. Then, the nurse called them to explain the project objective and their expected role and asked if they were willing to participate in our study. The nurse then shared the list of the participants that agreed to participate with the research team. As per ethics directives, the research team only received the list of those who agreed to participate.

After receiving the list from the FMPs of 32 PWD, a research assistant was in charge of contacting the potential participants to explain the project, their role as participants of the project, evaluate the PWD ability to consent, schedule a date for the consent form and the questionnaires for Phase 1.

Out of 32 PWD contacted, 29 agreed to participate in Phase 1 of our project. Of those, 25 had a caregiver present. All caregivers agreed to participate in phase 1.

For Phase 2:

- At the end of the appointment for Phase 1, the participants were asked if they were interested in being contacted for a semi-structured interview.
- The participants that agreed were contacted at another date for explaining the objective of Phase 2, evaluate the PWD ability to consent, and schedule a date of the interview.

Seven out of 17 dyads (PWD and caregiver) agreed to participate in the second phase. Reasons for refusal included change of residence (living outside Montreal), no longer interested in the study and impossibility of reaching them by phone.

### Data collection

#### Phase 1: Cross-sectional quantitative study

We collected information on sociodemographic characteristics of the patients and their caregivers (age, gender, educational level, spoken language, relationship with the care recipient, (not) living with the care recipient).

To evaluate the PWD’s cognition, we used the MMSE and MoCA tests [34]. To evaluate their needs, we used the *Camberwell Assessment of Need for Elderly (CANE)* for patients [35]; and the *Carers’ Needs Assessment for Dementia (CNA-D*) for caregivers [36].

To assess behavioral disturbance, we used the *Neuropsychiatric Inventory (NPI)* [33, 34]. The quality of life was evaluated using the *Quality of Life – Alzheimer’s Disease scale (QOL-AD)* [37]. See online Additional file 1 for more details on the tools.

All the questionnaires were available in English and French. The questionnaires were done by the second, third, and fourth author, between April 2019 and January 2020, at the place of preference of the participants. These authors were a nurse practitioner, and general practitioner, and a medical student, respectively. All of them have experience in research. We used the paper version of the questionnaires. This were then inputted into the database by the researcher who carried out the questionnaire.

#### Phase 2: Qualitative descriptive study

We carried out semi-structured qualitative interviews with PWD and their caregivers for exploring and understanding their experiences on services currently received at their FMP during the past 12 months, with special emphasis on dementia services, as well as the perceptions on those services for possible reasons for their met and unmet health and social care needs. The second, third, and fourth co-author conducted the interviews at the participants place of preference between April 2019 and January 2020 by pairs, depending on their availability. One of them was in charge of taking notes while the other facilitated the interview. The interviews were carried out either individually (one with the PWD and one with the caregiver) or together, depending on the preference of the participants. Interviews lasted between 11 minutes and 45 minutes, with an average of 28 minutes. Our semi-structured interview guide included questions about the beginning of their memory problems, their experience regarding their diagnostic process (including whether they received a diagnosis, by who and when), information received about the disease, resources available, and planning for the future (legal and financial advice, long-term care) as well as the questions on the unmet health and social needs highlighted by a patient or a caregiver in the quantitative phase to further explore possible causes and get the opinion on what could be done differently. Other questions included whether they had contact or not with a case manager and their experience with the care received at their clinic. Interviews were audio-recorded and transcribed verbatim.

### Data analysis

Data from phase one was analyzed using descriptive statistics using RStudio (1.0.153). Data from phase 2 was analyzed independently by second and third co-authors using inductive thematic analysis [38] in NVivo 12 who then met to identify convergence and divergence among researchers. Themes were refined and divergences were solved by consensus. The results were then shared with the first author.

### Integration of the quantitative and qualitative studies

**Fig. 1:**
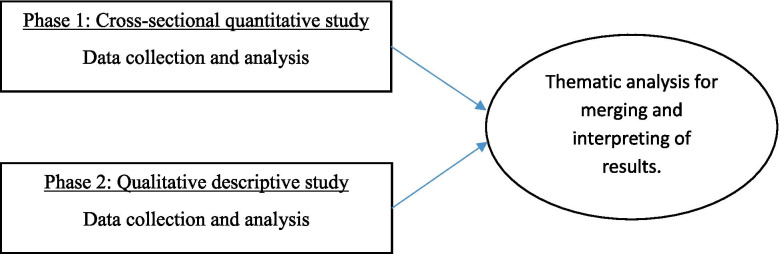
Study design based on a convergent mixed methods design

Results from the quantitative and qualitative phases were compared and contrasted and ultimately interpreted together [39]. The integration of the results from both studies was done using a narrative discussion approach [39] to analyze the collected data to identify the commonalities and required actions [40]. This approach allowed us to understand the phenomenon in more depth. A second and third authors identified the common themes of the qualitative study and shared with the first author. After the discussion and finalizing the themes, all authors together matched the unmet needs from the quantitative study with the themes of the qualitative study to propose the possible solutions that could be implemented to improve their needs and expectations (Fig. 1).

## Results

### Phase 1: Quantitative results

Our sample was composed of 29 PWD with a mean age of 79.1 (10.3; 60-108) years and 25 informal caregivers with a mean age of 71.3 (11.1; 41-90) years. The majority of PWD and caregivers had completed at least high school, and most of the participants were retired. Regarding the relationship of the dyads (n=25), 80% of them were a couple, 16% were a children of the patient, and 4% were friends (Table 1).

**Table 1:** Sociodemographic characteristics of PWD (n=29) and their informal caregivers (n=25)

	PLWD (29)	Caregivers (25)
Age (mean, SD, range)	79.1 (10.35; 60-108)	71.3 (11.1; 41-90)
Sex, male (number, %)	18 (62,1%)	4 (16%)
Highest level of education completed (number, %)		
Below high school	8 .6%)	7 (28%)
High school - Apprenticeship	11 (38%)	7 (28%)
College, CEGEP*, University or above	10 (34.5%)	11 (44%)
Work activity (number, %)		
Retired	28 (96.6%)	18 (72%)
Working part-time	0 (0%)	1 (4%)
Working full-time	1 (3.4%)	6 (24%)
Relationship with patient (number, %)		
Couple	20 (80%)	
Parent-child	4 (16%)	
Other	1 (4%)	

^*^CEGEP equivalent of the college in the Quebec education system

Table 2 shows the overall results of PWD and caregivers’ assesment tools. PWD were still in early stage of dementia with a mean MMSE score of 25.6 (4.5; 9-30) and mean MoCA of 21.3 (5.2; 8-28). PWD’s quality of life was scored in average 37.8 (5.7; 24-47) by the patient and 34.7 (6.9; 19-48) by the caregivers (the maximum score is 52). The low scores of NPI (5.34) indicates mild behavioral disturbances among PWD.

**Table 2:** Overall results of PWD and caregivers assessment (n=29)

Patient’s memory issues	Mean	SD	Range
Mini–Mental State Examination (MMSE)	25.66	4.56	9-30
Montreal Cognitive Assessment (MoCA)	21.31	5.21	8-28
**NPI**			
12 item NPI score	5.34	6.60	0-20
**QOL-AD**			
Scored by patient (n=29)	37.83	5.79	24-47
Scored by caregiver (n=25)	34.76	6.95	19-48
**CANE**			
Patient-reported met needs (n=29)	4,48	2,52	2-11
Patient-reported unmet needs (n=29)	0,55	0,91	0-4
Patient-reported total needs (n=29)	5,03	2,56	2-12
Caregiver-reported met needs (n=25)	5,08	2,55	2-11
Caregiver -reported unmet needs (n=25)	0,80	0,87	0-3
Caregiver -reported total needs (n=25)	5,88	2,77	2-12
**CNA-D**			
Caregiver reported number of moderate or serious problems (n=25)	3.12	2.35	0-7
Caregiver reported meet needs (n=25)	0.52	0.77	0-2
Caregiver reported unmet needs (n=25)	3.16	3.20	0-10

### Overall needs

The mean total number of needs reported by the patients was 5.03, of these 4.48 were met needs and 0.55 unmet needs. In contrast to the patients, the caregivers had only 0.52 met needs (3.16 unmet needs) (see Table 2).

### Specific patients needs

PWD’s self reported needs were in the following categories: memory, physical health, eyesight/hearing/communication, drugs, looking after home, money/budgeting as the main needs (figure 2 and 3 of online Additional file 1). However, most of these needs were met except for memory that was frequently rated by pairs of PWD and caregivers as unmet need. This was also reflected in the rating of the needs by a researcher (figure 4 of online Additional file 1).

### Specific caregivers needs

The needs of caregivers were lack of the emotional support, counselling, psychoeducation and psychotherapy as well as social worker support, printed information on dementia/treatment/services (figure 5, 6, 7 and 8 of online Additional file 1). They also rated high the frequency of physical and psychiatric illnesses but they were well addressed (figure 8 of online Additional file 1).

### Phase 2: Qualitative results

We conducted the interviews with seven pairs of PWD and caregivers. We identified three broad categories: attitude towards the memory decline, perception of the community health services and perception of the family medicine practice.

#### Attitude towards the memory decline

Both the patient and the caregiver noted a gradual decline of PWD’s memory that they frequently attributed to age. Despite having mentioned this tothe FPs, it was not often taken into account. However, the FPs would start an evaluation and the required referrals to specialized services when the memory decline became more obvious and interfering with the patient’s daily life:

*“ It took one year until the diagnosis […] I forget the keys, things in the shopping ...”* (patient)

Regarding the memory decline and diagnosis, participants indicated that they felt they did not receive enough information from the FP regarding this issue which led to lack of knowledge on the diagnosis.

“*The last thing the doctor said, was that (the patient) had an aging of the brain. He had seen the scan, he had done all that, and he found it was the aging of the brain […] I don’t know more than that. [The doctor] has not given us a diagnosis” (caregiver)*

#### Perception of the community health services

PWD and their caregivers often did not receive timely access to the community services when required , plus it was not tailored to their specific expectations and needs. In adddition, the regular change of the personnel who provided in-home services was another issue that required PWD and their caregivers to constantly adapt.

*“ [I] would like to have written material on the services” (caregiver)*

Those participants who had used community health services, had a case manager, who was in close communication with the caregiver. In one specific example, the case manager supported the caregiver with multiple changes of the home care staff since they were not trained to provide the appropriate care:

*“Let them send me a man who can take care of my husband. What are they sending me, a woman, but every time, it stresses me out […] I don't understand why they send incompetent people?*"

#### Perception of the family medicine practice

In contrast to the community services, PWD and their caregivers were in general satisfied with their FPs and nurses. They especially appreciated the FMPs’ nurses who spend more time with them and listen to their concerns. They also expressed a desire to have a more permanent doctor in the FMP as they found it difficult that their family medicine resident changed every two years.

*“[The nurse] would have more time in her hands than the doctor does. The doctor, I notice that she is running back and forth, back and forth. The nurse doesn’t do that.” (caregiver)*

Some participants indicated their family phsycian referred them for cognitive testing but did not follow-up on the results. Most of the participants stated that they needed more information regarding the prognosis, management at home, and how to access support services (including financial support services). If they had this information, it came from either a family member who worked in a health-related profession, or from other sources, such as a bank worker, but not from their primary care provider.

### Integration of quantitative and qualitative results (mixed methods integration)

The results integration enhanced our understanding of PWD and caregivers’ expectations in the context of the provincial Plan:

- The patients and their caregivers would like to have a contact person in the clinic, preferably a nurse
- The caregivers would like to know the diagnosis early on
- The caregivers would like to have a written care plan
- The caregivers would like to have more attention to their own emotionnal distress

## Discussion

Our empirical study describes the prevalence of met and unmet needs of PWD and their caregivers in the context of the Quebec Alzheimer plan. A study of the impact of the Quebec Alzheimer plan found that the evaluation of patients’ and caregivers’ overall needs were overlooked in more than 40% of the cases [27]. To evaluate met and unmet needs, we conducted a comprehensive assessement of the needs of PWD and their caregivers (quantitaive study) and then followed up by exploring and appreciating the experiences of the participants (qualitative study).

PWD rated their met needs highly (4.48) in contrast to their caregivers (0.52). A fewer unmet needs reported by PWD could be due to poor awareness of the problems or different priorities from their caregivers [41–45].

While our study confirms the findings of other studies on the most common unmet needs of the patients (e.g., information, memory) [46] and caregivers (e.g., information, caregiver burden) [46, 47], it also demostrated that the participants were satisfied with access to care and the medical services received in the primary care practices. Nontheless, our study shows that there are less unmet needs for PWD and caregivers compared to studies in other regions, such as the United States (most commont unmet needs were in the domains of safety and general care, and for caregivers, in the domains of referrals and education) [48, 49], China (unmet needs in the domains of social and environmental areas) [50] and Europe (unmet needs for PWD mainly in the social domain) [51]. The PWD and their caregivers expressed the confidence in their FPs but were open to the alternative such as nurse practitioners who are more available.

Caregivers often reported lack of the emotional support and counselling. While the community services to support the caregivers exist, this particular need continues to be prevalent [45, 52]. The explanation could be that frequently the caregivers opt to not use the services due to their unsuitability to their expectations [41]. Moreover, often a caregiver has FP different from the patient that adds a complexity to a comprehensive assessement of their needs.

Participants voiced their concerns about the change of their treating physicians (physician-residents) every 2 years that creates symptoms of uncertainty and anxiety [53]. However, given this reality of change every 2 years of the physician-residents, the participants were open to the idea that one person in the team can support the continuity such as a nurse clincian or practitioner [54].

The turnover of the personnel in the community services is another concern. The home care services should improve the continuity of homecare services such as to assign the same provider to the patients and their caregivers and to ensure the collaboration and good communication between family caregivers and staff from home services [55]. This can promote more consistency on patient centred care and more importantly, care based on a trusting long-term relationship.

Thus, our study provides a rich description of the needs of PWD and their caregivers that are either met partially or not at all. We would like to provide the following recommendations based on the voices of the participants:

- The family medicine practice should have a nurse practitioner as a key contact person for PWD and their caregivers. While the residents change in the teaching family practice every two years, the nurse practitioner could play a significant role in the continuty of care.
- The caregiver should receive a written information on the diagnosis, management, prognosis and available community services.
- The caregivers should have a separate appointment for an assessment of their needs.
- The psychological services of the community should extend their programs to caregivers support as well as to better understand and meet their expectations.
- The community services should focus on the recrutiment of personnel for permanent positions and assign them to the same patient-caregiver dyad.

Further research is needed to explore the needs of PWD depending on the stage of the disease, care received in the routine primary care vs university associated teaching facilities.

### Limitations

The main limitation of our study is the sample size. Due to unforseen circumstances (the COVID-19 pandemic) we were only able to recruit a limited number of dyads. Due to the restraints imposed by certain ethics committee on the recruitement of the vulnerable patients (in our case, patients with dementia), the recruitement strategy could consider local Alzheimer chapters. Given the sample size, we were not able to identify the needs according to the disease stage. Future studies should look at the differences in needs across the different stages and the evolution of the disease. However, the qualitative part of the study showed the saturation of the data across the clinical sites despite the small sample of the participants. Interviewing PWD and their caregivers together could omit the nuances in comparison to the individual interviews.

In terms of the study design, a quasi-experimental study on the needs of the patiens could be considered. Unfortunately, the Plan has already been implemented in all university-associated primary care unites to conduct a case-control study.

Our study focussed on the needs of patients with already established dementia diagnosis. Future studies could evaluate the needs of patients prior to the established diagnosis in primary care.

Another limitation is that the family medicine practices were the teaching sites that could limit the extrapolation of the results to other non-teaching sites.

## Conclusions

The Quebec Alzheimer plan with its focus on the involvement of FP and nurses in the primary care practices demostrated an overall success to meet the health needs of this vulnerable population. Our findings emphasize the importance to further tailor community services to better address the individual needs of PWD and their caregivers (e.g., a nurse as a contact person in the family medicine practice, the same healthcare provider of home care services).

## Supplementary Information

**Additional file 1.**

## Data Availability

The datasets used and/or analysed during the current study available from the corresponding author on reasonable request.
