# Supplementary material for: Needs of patients with dementia and their caregivers in primary care: lessons learned from the Alzheimer plan of Quebec
Source: BMC Fam Pract. 2021 Sep 15;22:186. doi: 10.1186/s12875-021-01528-3 (PMC8441033; doi:10.1186/s12875-021-01528-3)
Supplement: Supplementary file 1 — Additional file 1. [file 12875_2021_1528_MOESM1_ESM.docx]

**Online Appendix 1:**

Tools used for a cross-sectional study

To evaluate the needs, we used two validated tools: the *Camberwell Assessment of Need for Elderly (CANE)* for the needs of patients with dementia ^1^; and the *Carers’ Needs Assessment for Dementia (CNA-D*) for the needs of caregivers. ^2^ The CANE, which has been previously used to evaluate the needs of patients with dementia, ^3-5^ contains 24 areas of social, psychological, physical and environmental needs of patients. Needs are rated as no needs (score 0), met needs (score 1), unmet need (score 2) or unknown (score 9) for each area. Needs are met when a difficulty in a particular area is being provided for in such a way, that the person no longer feels its negative impact on his or her overall quality of life. Unmet needs are experienced when a person is not supported for a problem that occurs in a particular area or receives insufficient or inadequate support.

The CNA-D includes 18 problem areas with several possible interventions. ^2^ The severity of each problem area is rated on a three-point scale: no or mild problem; moderate problem; serious problem. The next step is to assess if the problem was addressed by using a four-point scale (CAN-D, version B): no need (intervention not needed and not received), unmet need (intervention needed, but not received), partially met need (intervention needed and received, but insufficiently), and met need (intervention needed and sufficiently received). ^2^ For each problem (mild, moderate, or serious), the interviewer has to assess which interventions are needed to ameliorate the problem. In any case – whether there is a problem or not – the interviewer assessed the interventions received by the caregiver (if requested).

The author of the CNA-D has granted permission to use the tool in this study (personal communication available on request).

To assess behavioral disturbance, we used the *Neuropsychiatric Inventory (NPI)* ^6-7^ which is based on the assessment of the caregiver’s responses about the frequency and severity of behavioral problems of the PLWD (e.g., anxiety, agitation). ^6^ Higher scores represent greater symptoms. The quality of life was evaluated using the *Quality of Life – Alzheimer’s Disease scale (QOL-AD).* ^8^ It consists of 13 items covering conceptual domains of quality of life in older adults. It uses simple and straightforward language; responses are structured in a four-choice format. Internal consistency reliability for the QOL-AD is 0.84. ^8^ The QOL-AD was rated by PWD about himself and then by the caregiver about his family member’s current situation. The total score can range from 13 to 52, with higher scores indicating better quality of life.

**Figure 1: Recruitment strategy**

No information on how many persons were contacted initially; only of those who agreed.

Final list: 32 PLWD

PLWD contacted: 32

PLWD who agreed to participate: 29

Caregivers who agreed to participate: 25

Dyads (PLWD + caregiver) interested in Phase 2: 17

Dyads contacted: 17

Dyads who agreed to participate in Phase 2: 7


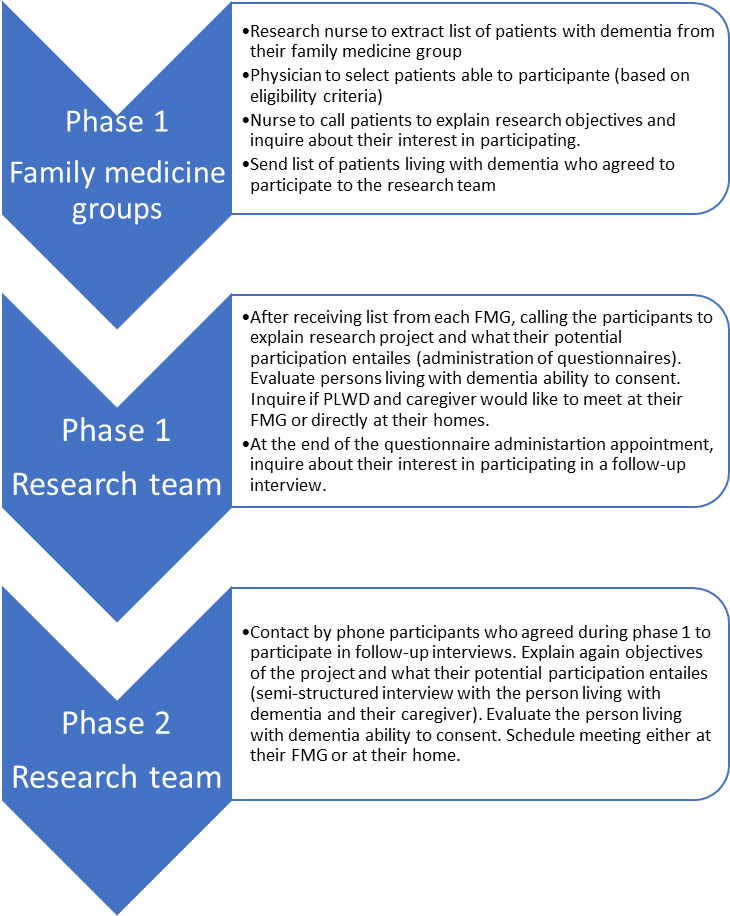


**Figure 2:** Camberwell assessment of Need for the Elderly: Patient’s rating of meet and unmeet needs (n=29)


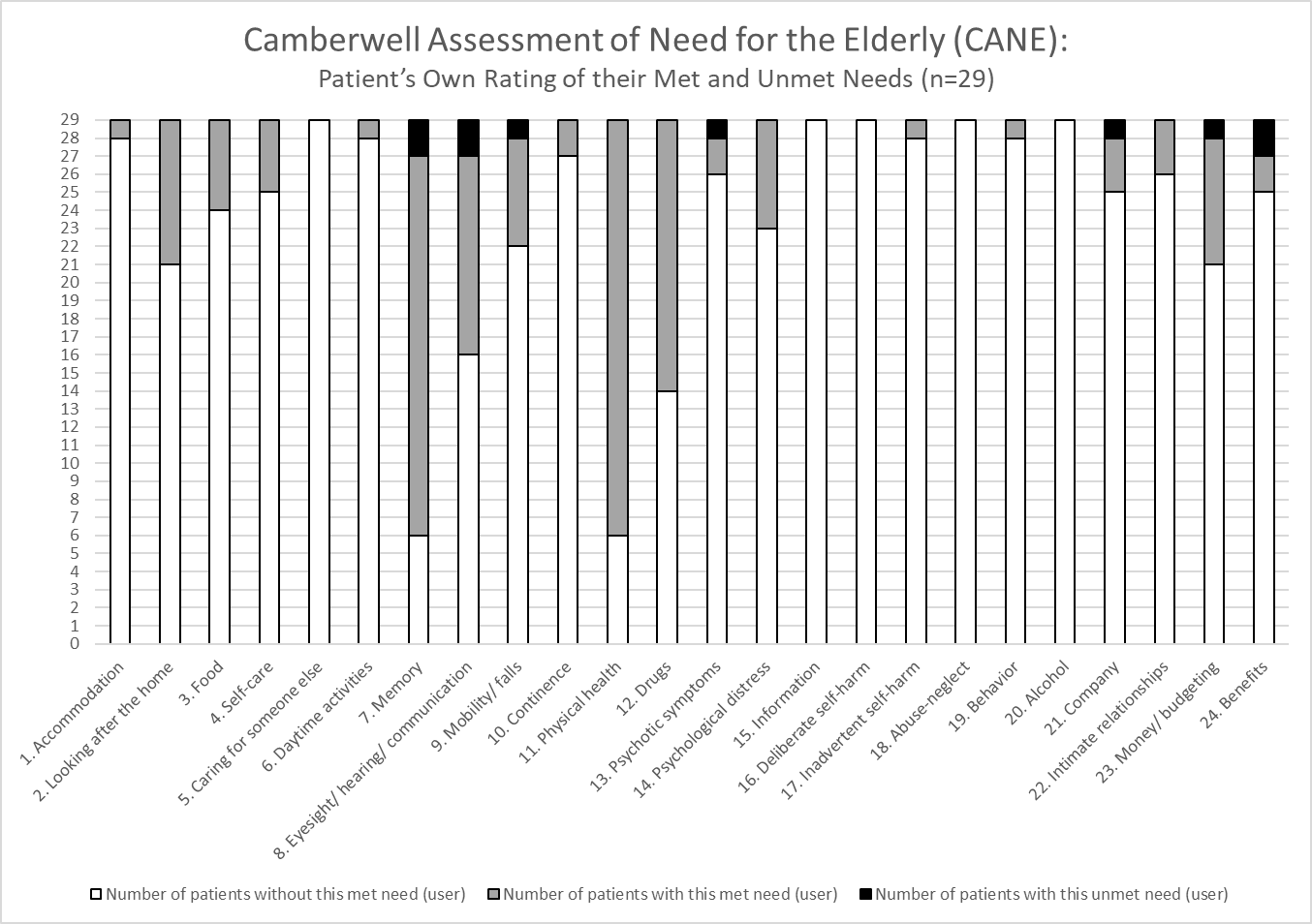


**Figure 3:** Camberwell assessment of Need for the Elderly: Caregiver’s rating of meet and unmeet needs (n=25)


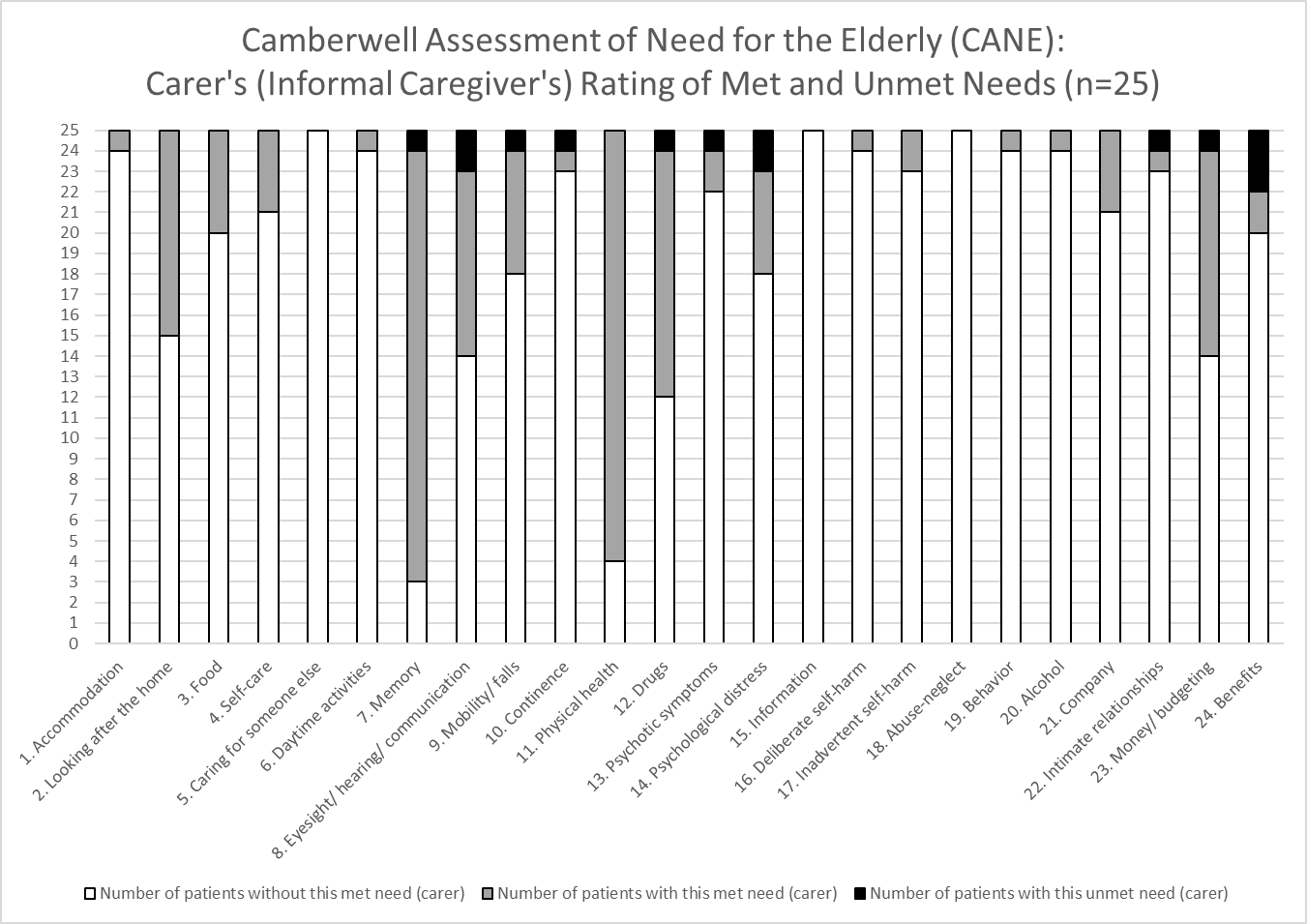


**Figure 4:** Camberwell assessment of Need for the Elderly: Researcher’s rating of meet and unmeet needs (n=29)


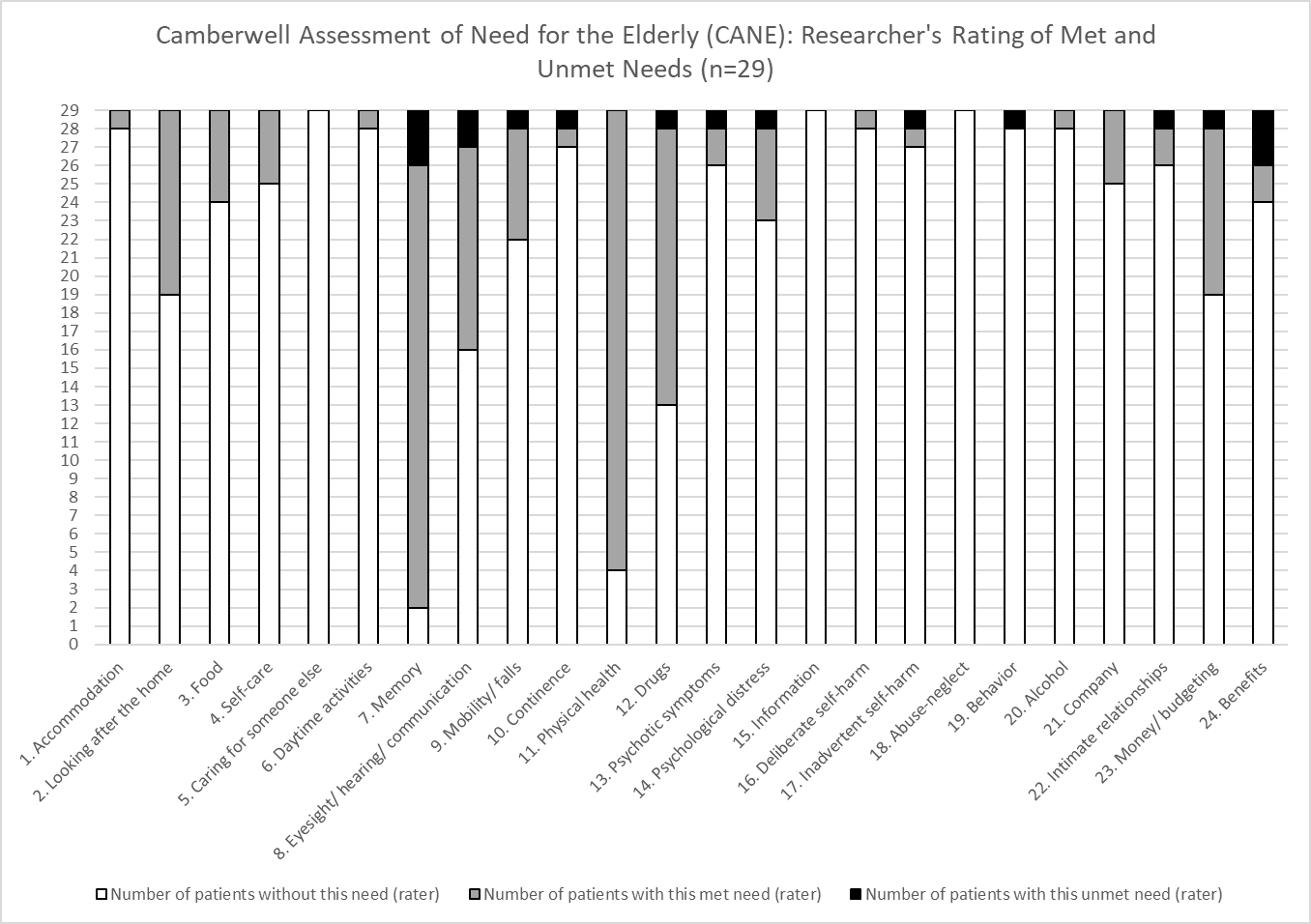


**Figure 5:** CNA-D: Meet and Unmet (or partially meet) needs of the informal caregivers as reported by the caregiver and the researcher (n=25) (Part 1 of 2)


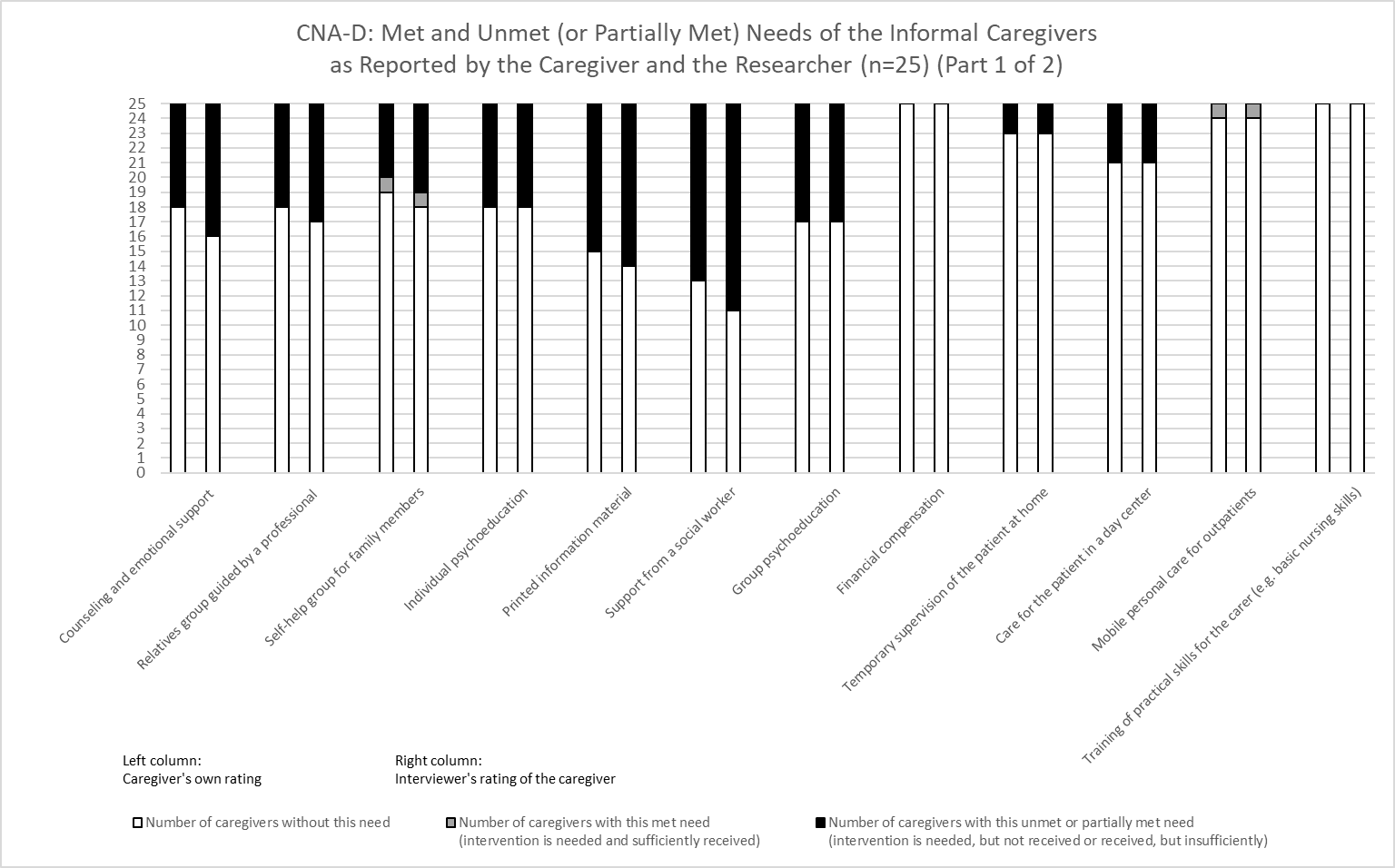


**Figure 6:** CNA-D: Meet and Unmet (or partially meet) needs of the informal caregivers as reported by the caregiver and the researcher (n=25) (Part 2 of 2)


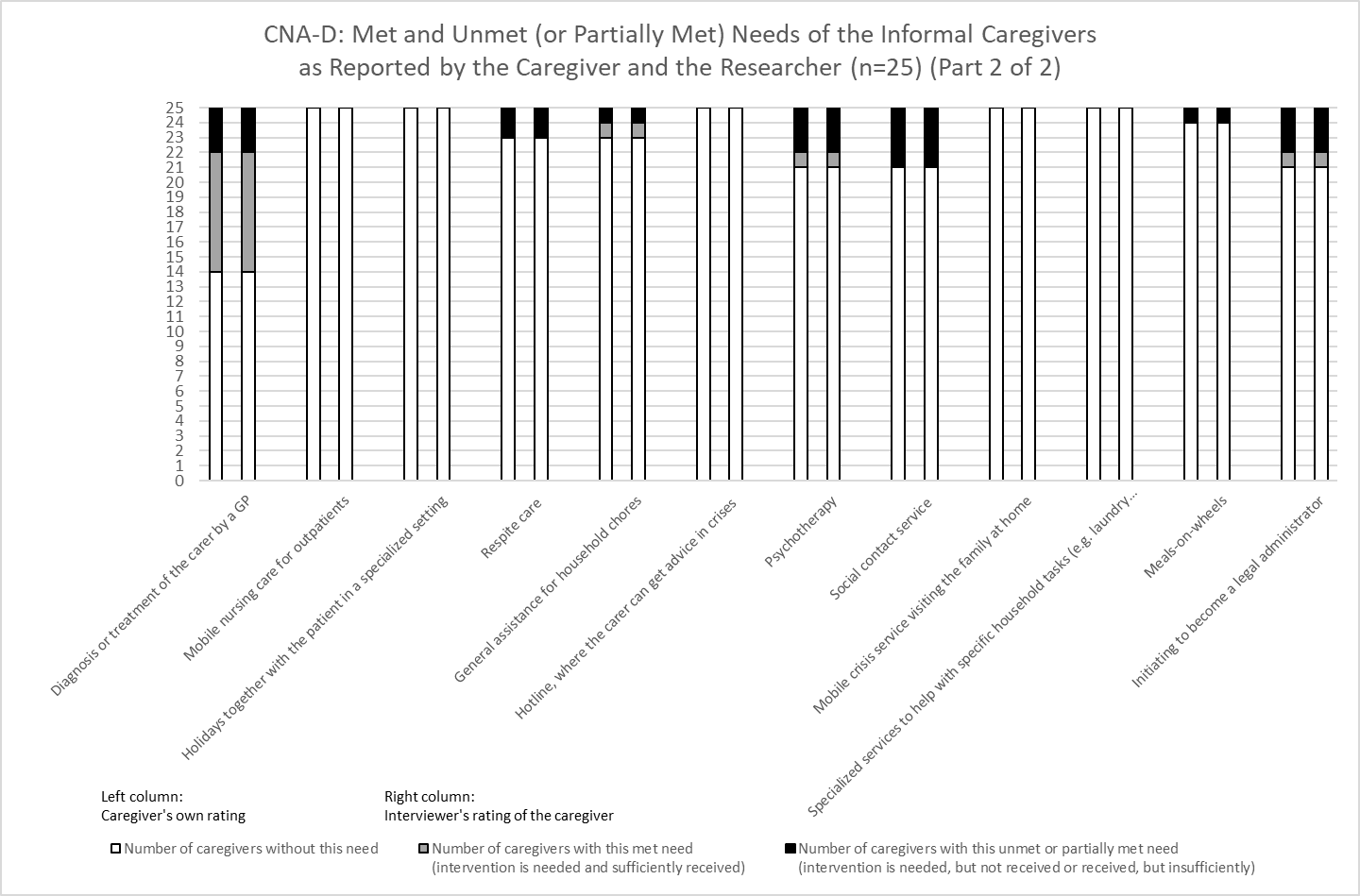


**Figure 7:** CNA-D: Number of Moderate and Serious Problems of the Informal Caregivers as reported by the caregiver and the researcher (n=25) (Part 1 of 2)


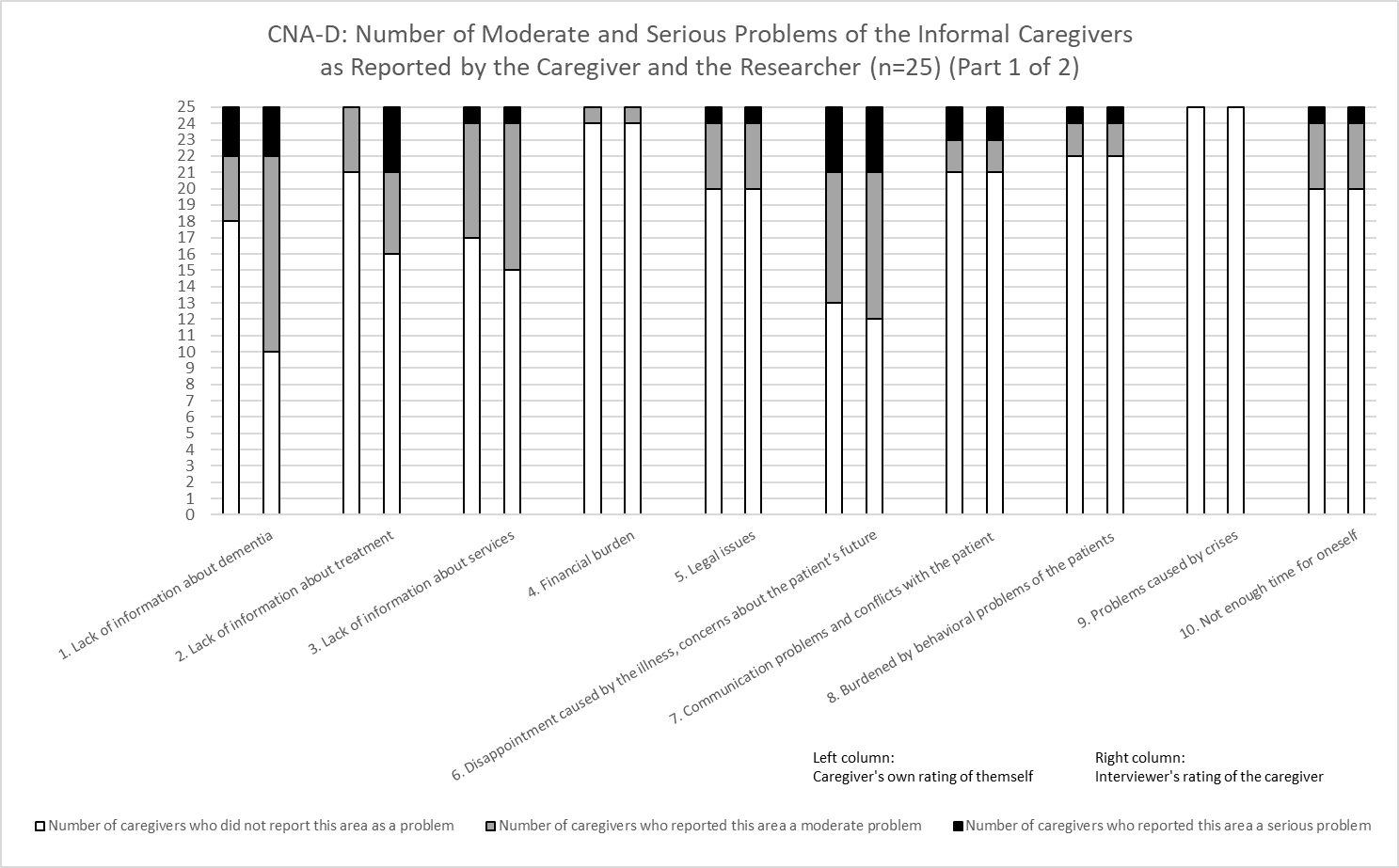


**Figure 8:** CNA-D: Number of Moderate and Serious Problems of the Informal Caregivers as reported by the caregiver and the researcher (n=25) (Part 1 of 2)


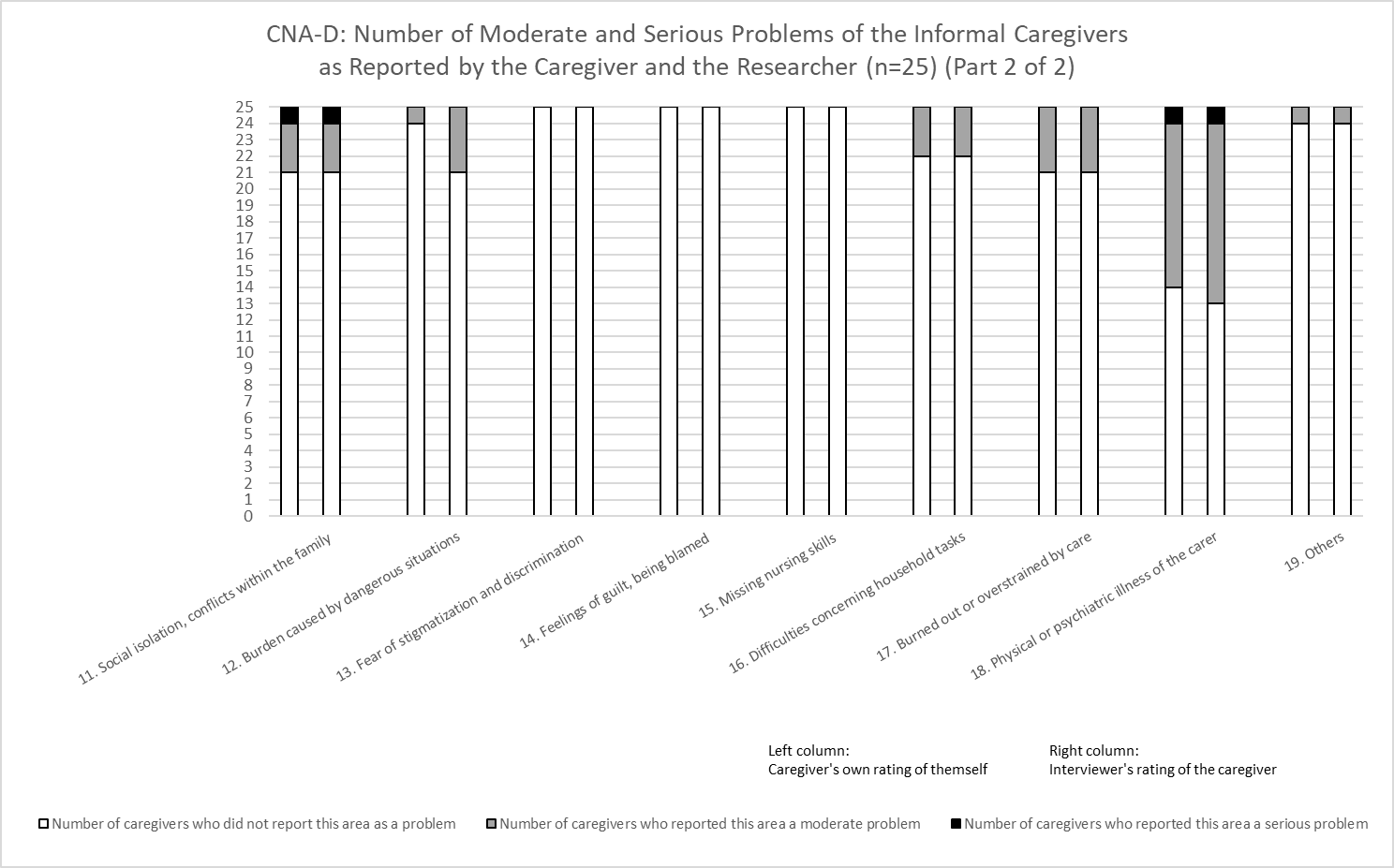


**References**

1. Reynolds T, Thornicroft G, Abas M, Woods B, Hoe J, Leese M, et al. Camberwell Assessment of Need for the Elderly (CANE). Development, validity and reliability. Br J Psychiatry. 2000;176:444-52.

2. Wancata J, Krautgartner M, Berner J, Alexandrowicz R, Unger A, Kaiser G, et al. The Carers' Needs Assessment for Dementia (CNA-D): development, validity and reliability. Int Psychogeriatr. 2005;17(3):393-406.

3. van der Roest HG, Meiland FJ, Comijs HC, Derksen E, Jansen AP, van Hout HP, et al. What do community-dwelling people with dementia need? A survey of those who are known to care and welfare services. International psychogeriatrics / IPA. 2009;21(5):949-65.

4. Miranda-Castillo C, Woods B, Galboda K, Oomman S, Olojugba C, Orrell M. Unmet needs, quality of life and support networks of people with dementia living at home. Health Qual Life Outcomes. 2010;8:132.

5. Miranda-Castillo C, Woods B, Orrell M. People with dementia living alone: what are their needs and what kind of support are they receiving? International psychogeriatrics / IPA. 2010;22(4):607-17.

6. Cummings JL, Mega M, Gray K, Rosenberg-Thompson S, Carusi DA, Gornbein J. The Neuropsychiatric Inventory: comprehensive assessment of psychopathology in dementia. Neurology. 1994;44(12):2308-14.

7. Moniz-Cook E, Vernooij-Dassen M, Woods R, Verhey F, Chattat R, De Vugt M, et al. A European consensus on outcome measures for psychosocial intervention research in dementia care. Aging Ment Health. 2008;12(1):14-29.

8. Logsdon RG, Gibbons LE, McCurry SM, Teri L. Assessing quality of life in older adults with cognitive impairment. Psychosom Med. 2002;64(3):510-9.
